# Exosome for mRNA delivery: strategies and therapeutic applications

**DOI:** 10.1186/s12951-024-02634-x

**Published:** 2024-07-04

**Authors:** Zoya Iqbal, Khurrum Rehman, Ayesha Mahmood, Maryam Shabbir, Yujie Liang, Li Duan, Hui Zeng

**Affiliations:** 1 Department of Orthopedics, Shenzhen Second People’s Hospital, The First Affiliated Hospital of Shenzhen University, Shenzhen, 518035 China; 2 Department of Allied Health Sciences, The University of Agriculture, D.I.Khan, Pakistan; 3 Department of Pharmacy, The University of Lahore, Lahore Campus, Lahore, Pakistan; 4 Department of Child and Adolescent Psychiatry, Shenzhen Clinical Research Center for Mental Disorders, Shenzhen Kangning Hospital, Shenzhen Mental Health Center, Shenzhen, 518020 China

**Keywords:** Exosomes, Targeted delivery, mRNA

## Abstract

Messenger RNA (mRNA) has emerged as a promising therapeutic molecule with numerous clinical applications in treating central nervous system disorders, tumors, COVID-19, and other diseases. mRNA therapies must be encapsulated into safe, stable, and effective delivery vehicles to preserve the cargo from degradation and prevent immunogenicity. Exosomes have gained growing attention in mRNA delivery because of their good biocompatibility, low immunogenicity, small size, unique capacity to traverse physiological barriers, and cell-specific tropism. Moreover, these exosomes can be engineered to utilize the natural carriers to target specific cells or tissues. This targeted approach will enhance the efficacy and reduce the side effects of mRNAs. However, difficulties such as a lack of consistent and reliable methods for exosome purification and the efficient encapsulation of large mRNAs into exosomes must be addressed. This article outlines current breakthroughs in cell-derived vesicle-mediated mRNA delivery and its biomedical applications.

**Graphical Abstract:**

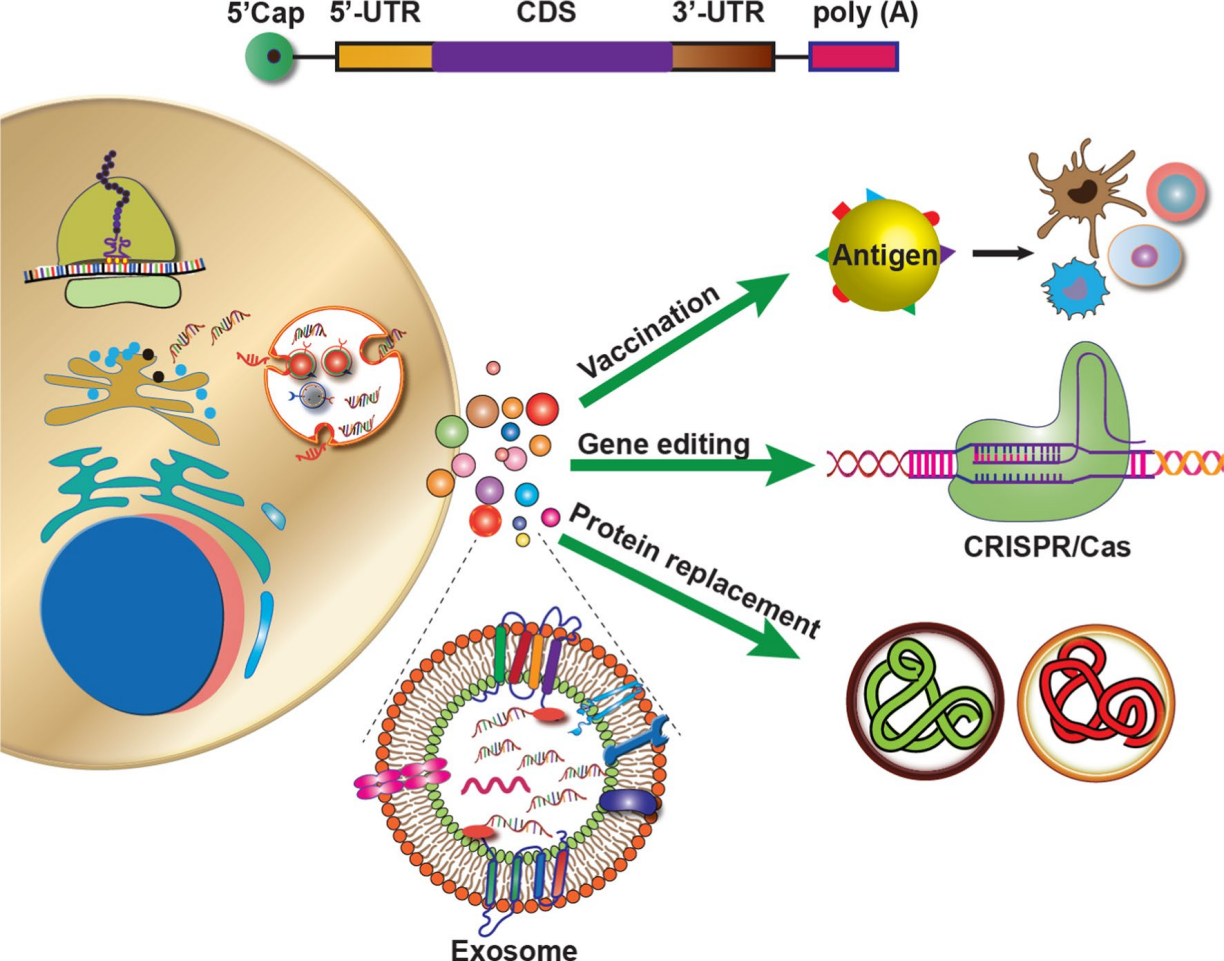

## Introduction

RNA therapeutics encompass various types of RNA, such as messenger RNA (mRNA), microRNA (miRNA), small non-coding RNA (sncRNA), ribosomal RNA (rRNA), small interfering RNA (siRNA), and long non-coding RNA (lncRNA) [1]. These RNA therapies have the potential to modify genomic or proteomic information through different mechanisms. Unlike traditional medications that merely address symptoms, the advancements in RNA-based medicines offer great promise in treating and preventing various human diseases by targeting the underlying pathophysiology. Nucleic acid-based treatments, particularly messenger RNA (mRNA), have played a pivotal role in recent advances in RNA-based vaccination, protein replacement therapy, gene editing, and cancer immunotherapy [2]. Recently, two mRNA vaccines targeting severe acute respiratory coronavirus type 2 (SARS-CoV-2) were licensed to prevent COVID-19 infection [3–6]. These developments have demonstrated the potential of mRNA to become a promising drug class. Although several RNA therapies have entered clinical trials, the FDA has only recently approved a few. Furthermore, the outcomes of RNA therapeutic clinical trials have been mixed, with some studies suggesting robust efficacy and others demonstrating toxic effects.

The main obstacle to the delivery of mRNA lies in its intrinsic instability and inability to penetrate the cellular lipid bilayer. Many efforts have been put on enhancing mRNA stability and lowering its immunogenicity [7, 8]. To ensure cargo protection and effective delivery into the cytoplasm of target cells, they must be enclosed in nanocarriers. Numerous researchers have also been attracted in developing mRNA delivery systems [9, 10]. Several nanomaterials have been reported to date, including lipid- and nano-based nanocarriers, for mRNA transport [11, 12]. Many obstacles still exist despite the rise in studies on delivery platforms, including toxicity and the precise distribution of mRNA to extrahepatic organs [13, 14].

In recent decades, exosomes as drug delivery systems (DDS) have shown remarkable advancements. Exosomes, membrane-enclosed nanoscale particles released by both prokaryotic and eukaryotic cells, are capable of transporting biological material such as proteins, lipids, RNA, and DNA. They have been isolated from numerous cell types, including normal and malignant cells [15]. These unique exosomes have attracted the attention of researchers in genetic engineering and drug development due to their potential use as delivery systems for various molecules. Exosomes have gained particular interest in the field of mRNA delivery due to their unique ability to cross physiological barriers, favorable biocompatibility, low toxicity, cell-specific tropism, and ability to evade the mononuclear phagocytic system [16, 17].

The ultimate goal of nanomedicine is the precise and targeted delivery of drugs to desired cells while avoiding harm to healthy tissues. The cargo-carrying capacity and selectivity of exosomes can be improved for greater therapeutic effects using various modified exosomal production technologies through chemical or genetic engineering. Engineered exosomes can deliver mRNA to specific sites, avoid immune system detection and early degradation, overcome biological barriers, and even control the release of the drug in response to a specific external stimulus [18, 19].

This review outlines the current developments and advancements in mRNA therapeutics, therapeutic payload loading approaches, the applications of cell-derived vesicle-mediated mRNA delivery, and surface grafting for improved cell selectivity. Furthermore, we critically evaluated the most recent findings in this field and outlined the key issues in employing exosomes as delivery platforms, along with some prospective solutions.

## Advances and challenges of mRNA therapy

### Transformative advances in mRNA therapies development

Over the past decade, therapeutic mRNAs have brought about a revolution in the field of drug development. Modified nucleotides in mRNA sequences can code for a wide range of proteins and thus treat various medical conditions, *e.g.*, genetic diseases, infectious diseases, cancer. Moreover, engineering synthetic mRNA sequences can enhance translation efficiency and robust protein production within target cells. In order to optimize the translation efficiency of mRNA molecules and achieve the desired protein expression, several key strategies and considerations must be taken into account [20]. These include enhancing stability and reducing immunogenicity through the incorporation of modified nucleotides like pseudouridine or 5-methylcytidine [21], increasing protein expression levels by optimizing 5' and 3' UTRs as they are crucial for translation [22], selecting the appropriate promoter for an expression vector to drive mRNA transcription i*n vitro*, and utilizing an efficient transfection method or delivery system for introducing mRNA into target cells [23]. Additionally, cytotoxicity can be minimized by optimizing transfection reagents, mRNA concentration, and transfection timing [24], and stabilizing elements can be added to mRNA sequences to ensure their stability within cells [25]. It is also important to note that post-translational modifications such as glycosylation and phosphorylation are necessary for proteins to function properly [26].

The analysis of a patient's genetic and molecular data plays a crucial role in identifying specific targets and optimizing the codon usage in mRNA sequences to specifically address the underlying disease mechanisms [27]. This flexibility allows mRNA therapies to be customized to meet the individual needs of each patient, which may offer better treatment outcomes. Furthermore, this optimization improves stability, translational efficiency, and target specificity.

Another advantage of mRNA therapies is their rapid development. Precision medicine technologies, *e.g.*, next-generation sequencing and high-throughput screening, permit researchers to rapidly identify potential therapeutic targets and create mRNA sequences that can regulate the expression of specific genes. This quick development process aids in rapidly translating scientific discoveries into clinical applications, potentially accelerating the delivery of novel therapies to patients. Different delivery systems ensure that mRNAs are efficiently delivered to target cells and achieve the required therapeutic effect [28].

### Applications and versatility of mRNA Therapy

mRNAs have opened up a new frontier in medicine, offering innovative therapies for various conditions due to their versatility and broad applications. Therapeutic mRNA can address various medical challenges associated with infectious diseases [29–31], tumor immunotherapy [32–35], cerebrovascular diseases, cardiovascular diseases, and rare genetic disorders [36]. By utilizing customized mRNA sequences, patients can obtain the precise genetic instructions required to produce functional proteins that may be defective/dysfunctional. This personalized approach allows treatments tailored to each patient's genetic mutations and needs. Various chemically modified mRNAs can have far-reaching effects when used for different therapeutic purposes, e.g., genetic reprogramming, immunotherapy, genome engineering [37–43], mRNA-based regenerative medicines [44–46], and protein replacement therapy [47–49] for various diseases [50].

### Translational challenges of mRNA

In addition to their great potential, the development and implementation of mRNA-based therapies present significant challenges that must be addressed [51]. One of the primary obstacles in mRNA-based therapeutics is the potential for immunogenic responses. Unmodified mRNAs can trigger inflammation and immune reactions when introduced into the body as foreign invaders, thus compromising efficacy and safety [52]. Chemical modifications of mRNAs are under investigation for their potential to reduce immunogenicity. Moreover, a therapeutic mRNA must be designed in a way to ensures that the intended proteins are produced in the targeted cells while avoiding off-target effects. It is possible to have adverse effects and safety concerns associated with the expression of proteins in non-target cells or tissues [53].

Another major challenge is delivering therapeutic mRNAs safely and efficiently to target cells and tissues. As a result of rapid degradation in the bloodstream, naked mRNA molecules can trigger immune responses. To protect and deliver mRNA to specific sites, exosomes, lipid nanoparticles, liposomes, and viral vectors are being utilized [40, 41, 54]. The advantages and disadvantages of different carriers in mRNA delivery are summarized in Table 1. It is complex to achieve tissue-specific targeting for diseases affecting multiple organs or tissues. Researchers are presently developing delivery systems that selectively target certain types of cells while sparing others.

**Table 1:** Pros and cons of different carriers in mRNA delivery [55–60]

Delivery systems	Advantages	Challenges
*Virus-like particles*	High transfection efficiency Long-term gene expression Protect mRNA from RNase degradation Efficient intracellular delivery	Low loading capacity Potentially carcinogenic Autoimmunogenicity High preparation costs Tedious production process
*Exosomes*	Immunocompatible if derived from an autologous source Natural features to deliver molecules to target cells Strong ability to cross biological membranes and high endogenous targeting potential Biocompatible and not easily cleared by the immune system	Limited clinical trials owing to uncertainties regarding exosomes Low drug loading and retention Rapid clearance from blood on in vivo administration Difficult to produce, isolate, and purify
*Lipid-based nanoparticles*	Active clinical research Protect mRNA from RNase degradation Efficient intracellular delivery of mRNA Diversity of lipid composition	Immunocompatibility may be problematic Dependent on the enhanced permeability and retention (EPR) effect Insufficient drug loading Difficult preparation
*Polymer-based nanoparticles*	Transient expression High packaging capacity Protect mRNA from RNase degradation Easy preparation	Polydispersity/self-aggregation Low gene delivery efficiency Low biodegradability and low efficacy Mostly non-degradable
*Polypeptides and cell-penetrating peptides (CPPs)*	Increase enzymatic tolerance Enhance cellular uptake Efficient targetability Low immunogenicity	Low delivery efficiency Availability of limited effective peptides Complex formulation Potential cytotoxicity
*Naked mRNA*	Easy to store and prepare Easy to scale up Cost-effective Simplified delivery	Prone to RNase degradation Rapid clearance from the system Immunogenic Low delivery efficiency
*Inorganic nanoparticles*	High packaging capacity Short transfection time Easy preparation Potential capability for targeted delivery and controlled release	Low gene delivery efficiency Instability Potential toxicity due to metal accumulation Poor biocompatibility and biodegradability
*Hybrid nanocarriers*	Reduce the toxicity of existing vectors Increase the transfection efficiency Improved the loading dose of DNA cellular uptake High transfection	Toxicity at very high dose Manufacturing complexities Stability issues

Moreover, synthesized mRNA is inherently unstable and can rapidly degrade without proper protection. The storage, transport, and administration of mRNA molecules must be carried out while maintaining their stability. To address these challenges, innovations are being explored in the fields of formulation and storage [61].

Furthermore, much complexity is involved in determining the optimal dosing regimen for mRNA therapies. Considering potential side effects and immune reactions is essential for balancing therapeutic efficacy and risk. Further, it is challenging to predict how long the treatment will last and what potential long-term effects will occur [62]. Besides, a developmental challenge is scaling up the manufacturing of therapeutic mRNAs. The production of mRNA products on a large scale requires specialized manufacturing processes to ensure consistent quality and purity [63].

## Exosomes as promising carriers of mRNA

### Exosome biogenesis and uptake

Exosomes, a subpopulation of extracellular vesicles (EVs), are produced by the endosome of cells, which are then released into the extracellular environment. Proteins, lipids, nucleic acids, and RNA can be transported by exosomes, which have a vital role in intercellular communication [64]. Multivesicular bodies (MVBs) and late endosomes are specialized compartments within the endosomal system that contain intraluminal vesicles (ILVs) [65]. MVBs are translocated to the plasma membrane through the cytoskeleton and microtubule network, fusing with the cell surface and undergoing cytokinesis, releasing ILV as exosomes [66]. However, some MVBs follow a degradation pathway and either fuse directly with lysosomes or autophagosomes, followed by lysosomal fusion [67]. Notably, MVBs are diverse, and whether their secretion and degradation pathways differ is uncertain. It is not clear whether specific markers or cargoes affect these pathways.

These ILVs selectively encapsulate specific proteins, lipids, and cytoplasmic components. When these ILVs are released, they become exosomes. ILV formation involves inward outgrowth of the endosomal membrane, a phenomenon first observed in transferrin receptor (TfR) vesicle secretion studies in mature reticulocytes [68]. Receptor-mediated endocytosis allows exosomes to target specific cells and supports cell communication, even over long distances. This can occur on lectins, adhesion molecules, and specific receptor-ligand partners. Specific cell types can mediate the endocytosis of exosomes through various lectins, *e.g.*, c-type lectin, galectin 5, and sialic acid on the vesicle membranes [69].

**Fig. 1:**
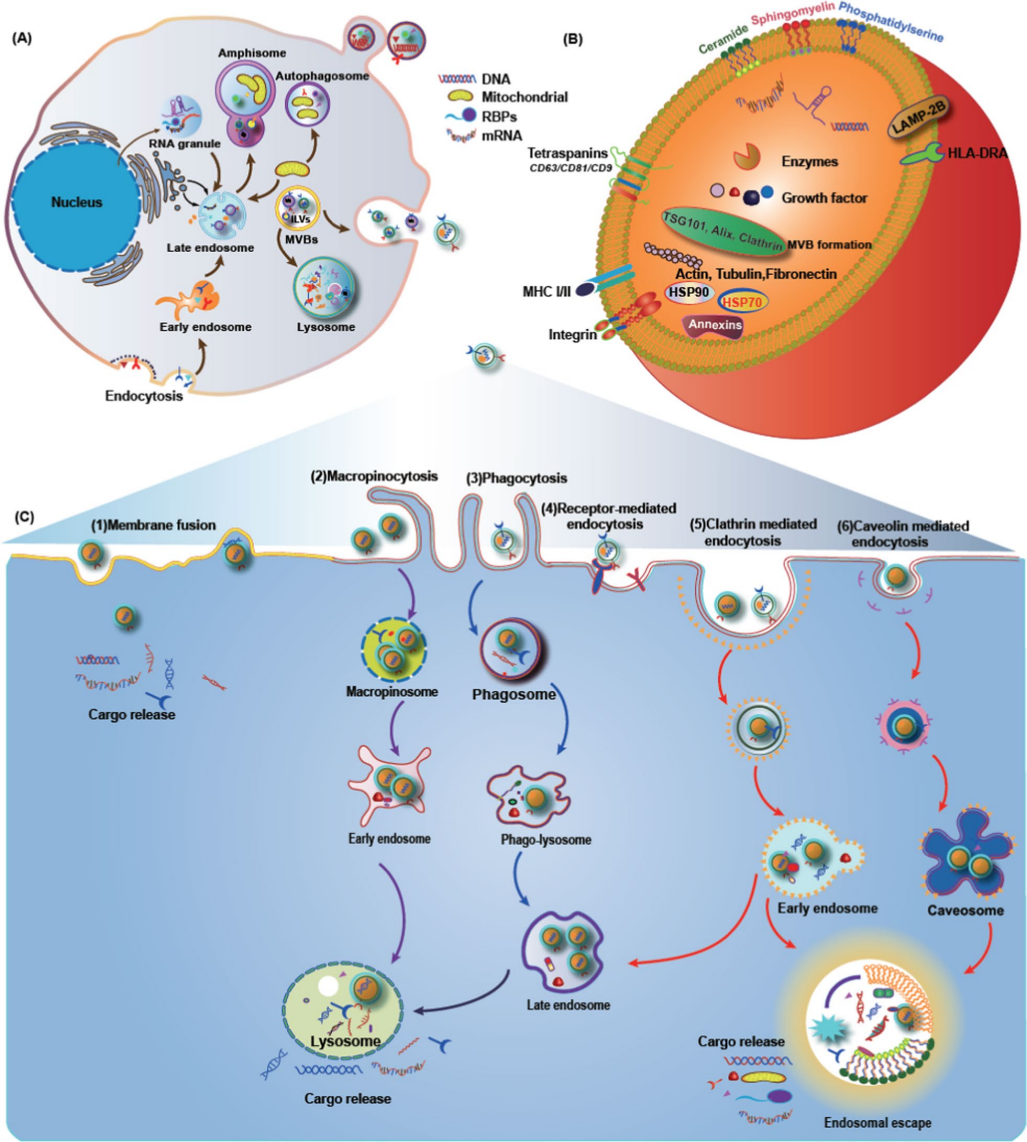
Biogenesis of exosoems and the mechanisms of uptake of exosomes involved by the recipient cell. **A** Early endosomes are produced from the invegination of the cell membrane, which mature into MVBs with ILVs inside. The inward budding of the endosomal membrane leads to ILVs formation. The fusion of the MVBs to the plasma membrane results in the secretion of exosomes extracellularly [71]. **B** Typical structure of exosomes containing functional DNA, proteins and RNA biomolecules surrounded by a lipid bilayer. **C** Several mechanisms have been reported for the uptake of exosomes, *e.g*., include micropinocytosis, phagocytosis, caveolae/raft-dependent endocytosis, receptor-mediated endocytosis, and direct fusion [72]. Adhesion molecules, including integrin, ICAM-1, LFA-1, CD81, and CD9 on the vesicle membranes, also play important roles in the binding and uptake of exosomes. Moreover, several receptor-ligand interactions, such as heparin sulfate proteoglycans-fibronectin, TIM receptors-phosphatidylserine, and epidermal growth factor receptor (EGFR)-epidermal growth factor (EGF), mediate the exosome endocytosis [73]

The detailed mechanisms regulating cargo sorting and ILV formation involve three pathways: the endosomal sorting complex required for transport (ESCRT)-dependent pathway, the ceramide pathway, and the tetra-transmembrane protein pathway. The ESCRT-dependent pathway is better characterized than the other two pathways and involves four protein complexes: ESCRT-0, -I, -II, -III and more than 20 proteins [70]. In addition, the importance of mechanisms involving tetra transmembrane proteins and lipids should not be overlooked, as they contribute to our understanding of the kinetics of exosome production and release. Figure 1 exhibits the biogenesis of exosomes and the underlying mechanisms of uptake of exosomes by different cells.

### Exosomes in mRNA delivery

Exosomes are naturally produced by various cell types, including immune cells, cancer cells, and stem cells [74]. The ability of exosomes to encapsulate RNAs, particularly mRNA, is a significant advantage for mRNA delivery. As a drug delivery vehicle, exosomes play a fascinating and promising role in delivering mRNA. Exosomes provide a protective microenvironment for the loaded cargoes. The lipid bilayers of exosomes protect mRNA from degradation by extracellular enzymes, ensuring its integrity and stability during transit to target cells [75].

Furthermore, the biofilm coverage of exosomes allows them to avoid degradation by macrophages, hence prolonging their circulation time [76]. Notably, exosomes exhibit remarkable stability *during* blood circulation, permitting them to transport mRNA over long distances in vivo*,* regardless of normal or pathological conditions. Moreover, exosomes also can cross biological barriers and migrate to organs that lack a blood supply. The exact mechanism by which exosomes cross these barriers is not yet fully understood. However, it is believed to involve interactions with specific receptors or transporters present on the barrier cells [77–79]. Further research is needed to elucidate the detailed process. Apart from their capability to interact with recipient cells and facilitate uptake, exosomes can interact with their cargoes and facilitate their loading [80]. Exosome cargoes can be released directly into the cytosol by the fusion of the exosomes with the target cell's plasma membrane.

Immunogenicity is a critical consideration in mRNA therapy, and exosomes offer a potential solution. Compared to synthetic delivery systems, exosomes administered in vivo result in fewer immunogenic reactions, toxicity, and adverse effects due to their biocompatible nature. This makes exosomes a safer and more efficient option for mRNA delivery [81].

One of the key benefits of exosomes is their ability to express surface proteins and receptors of their donor cells, allowing them to target recipient cells within the same tissue effectively. For example, lung spheroid cell-derived exosomes can deliver vaccines directly to lung tissue [82]. In addition, exosomes can be genetically engineered, chemically surface modified or enzymatically engineered to introduce peptides and proteins to enhance their targeting ability [42, 83–86]. Using mRNA therapy increases precision and specificity [87].

Preclinical studies have demonstrated the benefits of exosome-based mRNA therapies, which are moving toward clinical trials [88]. In clinical applications, exosomes are a promising vehicle for delivering mRNA. The use of exosomes as mRNA carriers is currently being investigated in various fields, including gene therapy, cancer treatment, regenerative medicine, and vaccination [89]. Although exosomes present numerous advantages, the clinical application of exosomes is still in its early stages as various challenges hinder their applications. Table 1 presents a summary of the advantages and disadvantages of different carriers in mRNA delivery.

## Strategies for mRNA loading into exosomes

Exosomes have served as an efficient method for mRNA delivery in gene therapy compared to traditional nucleic acid delivery vectors [90]. However, using exosome-based mRNA delivery systems has several limitations, such as the technical difficulties in generating exosomes loaded with RNA [91]. Encapsulating large RNAs, *e.g.*, mRNA, into exosomes is also a technical challenge [92]. Moreover, only a few cellular sources can release exosomes sufficiently for clinical applications [93]. Traditional cellular cultures cannot produce the required quantities to meet clinical dosage requirements. Furthermore, large numbers of cell cultures must be incubated over several days before the desired gene-enriched vesicles can be obtained.

Two primary approaches can accomplish the loading of specific cargoes into exosomes. The first method is endogenous loading, in which donor cells are engineered to contain the desired cargo by utilizing signature sequences and transfection techniques [15]. This ensures that the exosomes secreted by these cells naturally contain the desired RNA. The second approach, exogenous loading, involves purifying exosomes from various sources and loading the RNA into isolated exosomes in vitro. This can be accomplished by electroporation, chemical transfection reagents, or hybrids of exosomes-liposomes and cellular nanoporation [94, 95].

### Pre-loading methods

#### Passive pre-loading methods

Recent advances in loading mRNA into exosomes have involved introducing mRNA-encoded DNA into donor cells. After a transfection period of 24–48 h, the medium containing the exosomes is collected, and the transcribed mRNA from the plasmid is obtained. This technique is highly efficient because the donor cells naturally package the desired mRNA into the exosomes. Several studies have utilized this method to load various types of mRNA into exosomes [96].

The low-density lipoprotein receptor (Ldlr) mRNA was successfully encapsulated into exosomes by inducing overexpression of the plasmid in donor cells. Transfecting the cells with this plasmid resulted in a more than 100-fold increase in Ldlr mRNA levels compared to cells transfected with the control plasmid. As a result, the secreted exosomes had a similar increase in mRNA cargo [96].

To load Bone morphogenetic protein 7 (Bmp7) mRNA into exosome, parent cells were transfected with an overexpressing plasmid, followed by CP05-TK-mPEG stealth coating. This system, called SmartExo@Bmp7, demonstrated therapeutic advantages by specifically delivering Bmp7 mRNA to the adipose tissue (OAT) of obese mice, inducing browning induction [97].

In conclusion, the main advantage of passive pre-loading methods is their high efficiency due to the natural packaging of desired mRNA into exosomes by the donor cells. However, there are technical challenges in loading large mRNAs into nanometer-sized exosomes [98].

#### Active pre-loading methods

Active pre-loading methods involve loading mRNA into exosomes through the transfection of donor cells with two types of plasmids. One plasmid encodes fusion proteins consisting of mRNA-binding elements, while the other plasmid encodes exosome-rich proteins, *i.e.*, the surface markers CD63 and CD9 or the cytoplasmic protein Hspa8 [99].

Another approach is to actively package mRNA using conserved sequences of exosome-rich RNA (eRNA). These eRNAs may possess a specific common sequence that can act as a cis-element, allowing the targeted delivery of RNA to exosomes. Researchers have identified three potential signature sequences, providing clues for future therapeutic purposes to selectively target candidate mRNAs to exosomes [100].

**Fig. 2:**
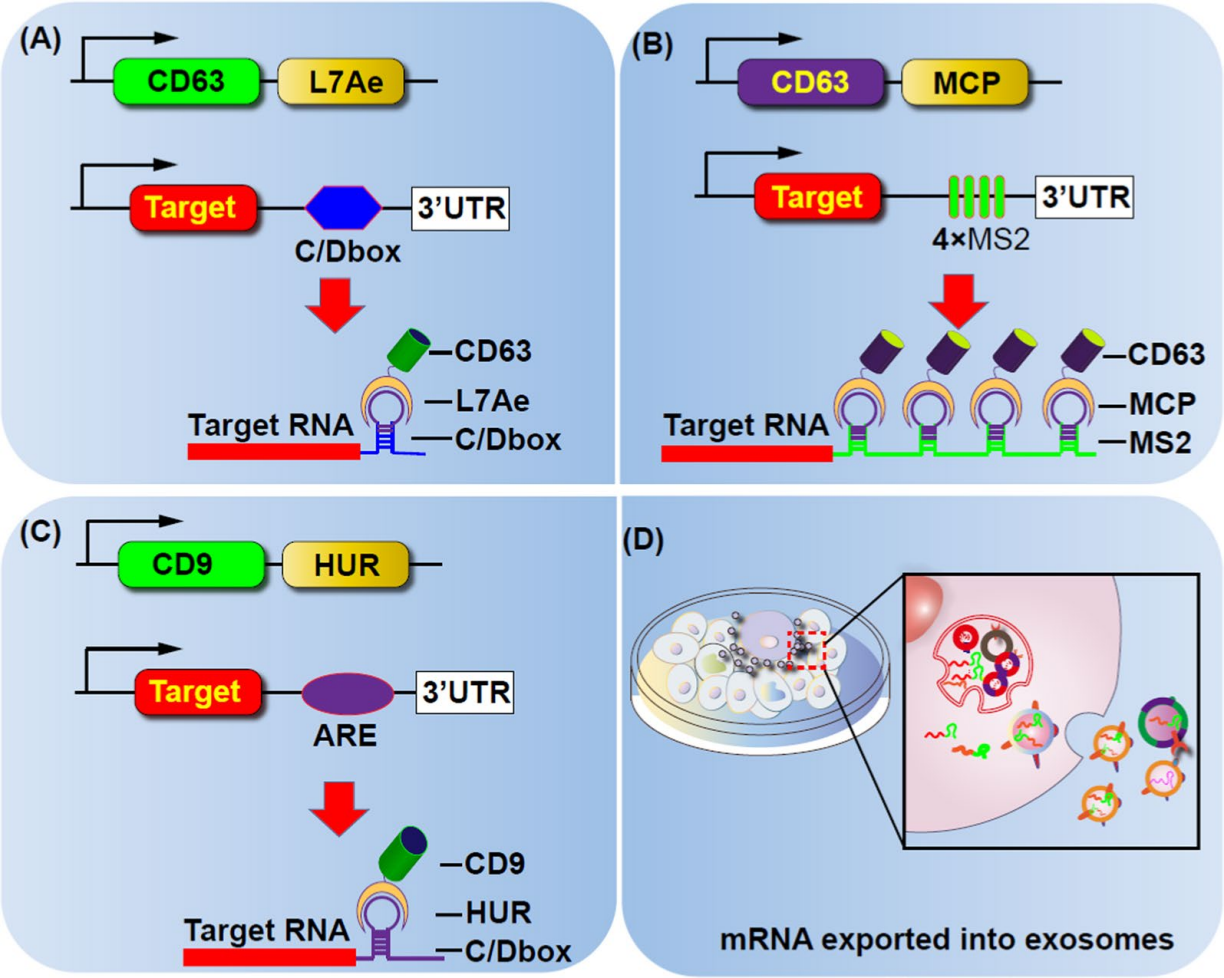
Schematic representation of programmable exosomes for mRNAs loading

**Fig. 3:**
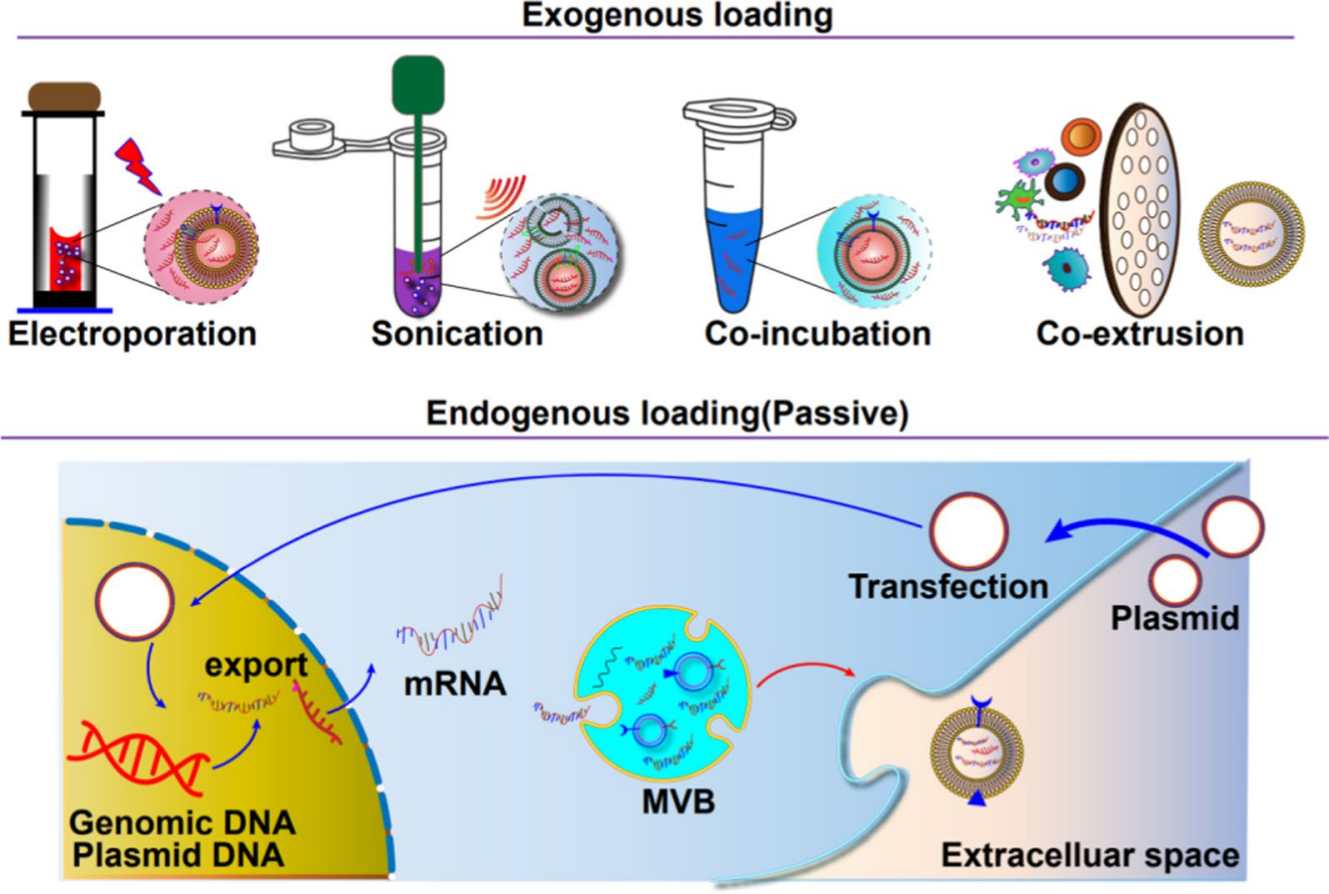
Different strategies for loading cargoes into exosomes

In order to improve the mRNA packaging efficiency, they took the advantage of the ancient ribosomal protein L7Ae uniquely binds a box C/D RNA. The L7Ae were fused with the C-terminus of CD63 and the C/Dbox repeat structure were inserted into the 3′-untranslated region of the target gene, so that the transcript of the target gene could be actively packaged into exosomes through the interaction between the L7Ae protein and C/D box (Fig. 2A)[101]. In another similary study, outer membrane vesicles (OMV) derived from bacteria was genetically engineered with L7Ae and Listeria monocytogenes hemolysin O (OMV-LL) to efficiently adsorb mRNA antigens labeled with adsorb box C/D sequence through L7Ae binding. These OMV-LL-mRNA complexes successfully delivered mRNA to DCs, leading to a significant inhibition of melanoma progression [102]. Moreover, targeted and modular EV Loading (TAMEL) system employs the fusion of EV-rich proteins (*e.g*., Lamp2b, CD63, or Hspa8) to MS2 phage shell proteins.This platform fuses EV-rich proteins (*e.g.*, Lamp2b, CD63, or Hspa8) with MS2 phage shell proteins. Homologous MS2 stem-loop sequences were integrated into the mRNA cargo to facilitate mRNA binding and encapsulation into the EV. This approach significantly increased the RNA cargo loading up to sixfold. Applying this method to exosomes expressing vesicular stomatitis virus glycoprotein (VSVG) resulted in a 40-fold enrichment of cargo RNA load (Fig. 3B) [99].

Engineered exosomes can also cross the blood–brain barrier (BBB) and deliver mRNA to treat neurodegenerative diseases. Peroxidase mRNAs fused to C/D cassettes were designed for effective exosome targeting. The designed exosome carrying RVG-Lamp2b was able to efficiently cross the blood–brain barrier (BBB) and deliver the catalase mRNA, resulting in a reduction of 6-OHDA-mediated neuroinflammation in a mouse model of Parkinson’s disease [101, 103].

Selectively targeting candidate mRNAs to exosomes can induce browning of white adipocytes and demonstrate anti-inflammatory effects to treat metabolic disorders. In this strategy, a two-part DNA aptamer was designed for efficient loading of mRNA. The single-stranded portion of the DNA aptamer recognizes the AUG codon of the specific mRNA, while the double-stranded part has elements identified by the CD9-ZF motif-engineered exosome system. This strategy efficiently delivered loaded Pgc1α mRNA to induce the browning of white adipocytes. Similarly, the system demonstrated an effective anti-inflammatory effect in an inflammatory bowel disease (IBD) mouse model upon the delivery of interleukin 10 mRNA [104]. To enhance RNA loading into exosomes, fusion proteins were created that bind the exosome membrane protein CD9 to RNA-binding proteins. The RNAs of interest naturally contain or are modified to contain the binding elements of the fusion proteins. CD9 was successfully fused to HuR, an RNA-binding protein that interacts with miR-155 with high affinity. In exosome-producing cells, the CD9-HuR fusion protein effectively enriched exosomes with miR-155 when miR-155 was overexpressed. Moreover, miR-155 encapsulated in these exosomes can be efficiently delivered to recipient cells and target endogenous molecules (Fig. 2C) [105].

Exosomes were engineered by combining zinc finger (ZF) motifs with CD9. In addition, a DNA aptamer was also designed that recognizes and assists in encapsulating the target mRNA into the CD9-ZF-engineered exosomes. Electroporating the DNase before delivery of the exosomes to the recipient cell could degrade the binding between the DNA aptamer and the target mRNA, leading to controlled and release of the mRNA [104].

Furthermore, exosomes can be designed for CAR T cell production and cancer immunotherapy. Engineered exosomes deliver chimeric antigen receptor (CAR) mRNA and anti-CD3/CD28 single-chain variable fragments (scFv) for the ex vivo production of CAR T cells with tumor-killing ability. These findings demonstrate the prospective application of engineered exosome delivery platforms in converting primary T cells into CAR T cells directly, furnishing a new approach for in vivo CAR T cell production [106].

Although active pre-loading methods have advantages in selectively targeting candidate mRNAs and increasing RNA cargo loading, they require the transfection of donor cells with specific plasmids, which may be more labor-intensive and time-consuming compared to passive pre-loading methods.

### Post-loading methods

#### Electroporation

Electroporation has been the primary method utilized for loading RNAs into exosomes. It involves the introduction of various molecules, such as siRNAs, miRNAs, and mRNAs, into purified exosomes [107]. Previous studies have demonstrated that electroporation can load approximately one-fifth of Cas9 mRNA into EVs derived from erythrocytes [92].

It is worth noting that electroporation-based transfection of nucleic acids into exosomes is not as potent or efficient. The process requires purification and isolation of exosomes before and after transfection, which involves high-speed centrifugation that can potentially damage exosomes and compromise sample quality. In addition, repetitive purification steps may cause the loss of exosomes [108]. Thus, electroporation is unsuitable for loading certain types of RNAs, such as miRNAs, shRNAs, and mRNAs, into exosomes [109].

#### Cellular nanoporation (CNP)

Cellular nanoporation (CNP) is a technique that involves placing donor cells on a nano-engineered silicon chip and using electrical stimuli to inject synthetic DNA into the donor cell. The CNP technology increases the exosome yield and the mRNA amount within the exosomes [110]. Compared to exosomes produced endogenously without involving external stimuli, CNP-treated exosomes from cells of the same origin have an increased mRNA encapsulation efficiency of 3–4 folds. In addition, this strategy increased mRNA loading in exosomes by 100-fold compared to conventional electroporation methods. This CNP approach allows using exosomes as potential carriers of mRNA for applications requiring transcriptional manipulation.

Exosomes generated from human dermal fibroblasts via CNP are loaded with mRNA encoding ECM α1 type I collagen (COL1A1). These exosomes promoted the collagen grafts formation and reduced the formation of wrinkles in the photoaging dermal collagen depletion mice model. In addition, intradermal delivery of exosome loaded with mRNA using a microneedle array improved and prolonged collagen synthesis and replacement in the animal dermis [111]. Thus, this system has the potential to be a protein replacement therapy for treating photoaged skin.

#### Transfection with specific reagents

Transfection is a common technique of loading proteins, nucleic acids and peptides into exosomes. This involves transducing specific plasmids into cells using transfection reagents, which ectopically express the required biomolecules and are packaged into exosomes. Although liposomal structures can easily load mRNA and DNA, they are not efficient in delivering these therapeutic substances to target cells [112]. On the other hand, exosomes are highly efficient in delivering cargo to cells due to their unique transmembrane proteins that promote cell attachment and facilitate endocytosis [113]. Lipofectamine, HiPerFect, and ExoFect are commonly used transfection agents. Moreover, a loading reagent called REG1 has been used to encapsulate mRNA into exosomes post-separation [114].

#### Exosome-liposome hybrid system

By associating mRNA with cationic liposomes and incubating these liposomes with exosomes, the exosome-liposome hybrid system can be engineered to efficiently deliver mRNA to cells [115]. In cases where liposome-mRNA complexes do not interact with exosomes, an isolation step may be necessary. These exosome-liposome hybrids have been shown to form structures larger than 200 nm [113], and ultrafiltration can separate them from smaller mixtures of exosomes and liposomes. Researchers have successfully synthesized folate-modified exosome-liposome hybrid nanoparticles loaded with ALKBH5 mRNA, which showed significant inhibition of colorectal cancer progression in tumor models by modulating the ALKBH5/JMJD8/PKM2 axis and hampering glycolysis [116].

Exosome-liposome hybrids have also been used to successfully deliver Antares2 mRNA into target cells. This involves coating mRNA with a polycationic lipid and incubating it with exosomes. This approach successfully developed a multiplexed COVID-19 vaccine based on mRNA [117]. Nonetheless, a major drawback of this approach is the challenge in separating the exosome from the transfection reagent, making it uncertain whether transfection results from the reagent or the exosome itself.

Exosomes play a vital role in intercellular communication by transferring proteins and RNA between cells. Elucidating the mechanisms by which exosomes load and deliver cargo molecules is essential for understanding the biological functions of exosomes and developing exosome-based therapies. While some of the motifs that regulate the encapsulation of biomolecules into exosomes have been identified, the rules that generally control cargo loading and transportation are still poorly understood. Figure 3 illustrates different approaches, both exogenous and endogenous, for loading cargo into exosomes.

## Exosome modification for mRNA targeted therapy

Exosome-mediated intracellular delivery of nucleic acids is an emerging and promising approach for genetic therapy. Nonetheless, a major obstacle to its clinical application is the non-specific uptake of exosome by non-target tissues, leading to off-target effects. To address this challenge, researchers are exploring ways to modify exosome contents, including their proteins, lipids and nucleic acids, in order to enhance their therapeutic potential. These engineered exosomes can be used as carriers for targeted delivery of biomolecules. Two main approaches can be used to enhance the targeting ability of exosomes for specific applications: direct modification of exosomes by chemical means or, indirect manipulation of the donor cells through genetic engineering techniques [89, 103, 118–120]

### Chemical modification of exosomes

Chemical modification of exosomes was carried out to target GL261 gliomas and U87 glioblastomas. The exosomes were functionalized with CREKA and CDX peptides specifically for each type of tumor. By modifying the exosome CD47 with these peptides, the targeting ability was improved. Moreover, when PTEN mRNA was additionally modified with the CDX peptide, the resulting exosomes exhibited a twofold increase in accumulation in mouse U87 gliomas, leading to prolonged survival. Similarly, modification of PTEN mRNA with CREKA peptide resulted in a 1.5-fold increase in accumulation in mouse U87 gliomas and improved survival [110].

RBCEV (red blood cell-derived extracellular vesicles) were modified by binding to nanobodies that targeted α-EGFR and α-HER2. This modification aimed to enhance the specific uptake of RBCEVs by target cells expressing these receptors. The surface functionalized RBCEVs were loaded with mRNA and paclitaxel, facilitating the targeted delivery of these cargo molecules to the desired target cells. In addition, the system demonstrated specific uptake of RBCEV by lung tumor cells expressing EGFR in vivo [114].

**Fig. 4:**
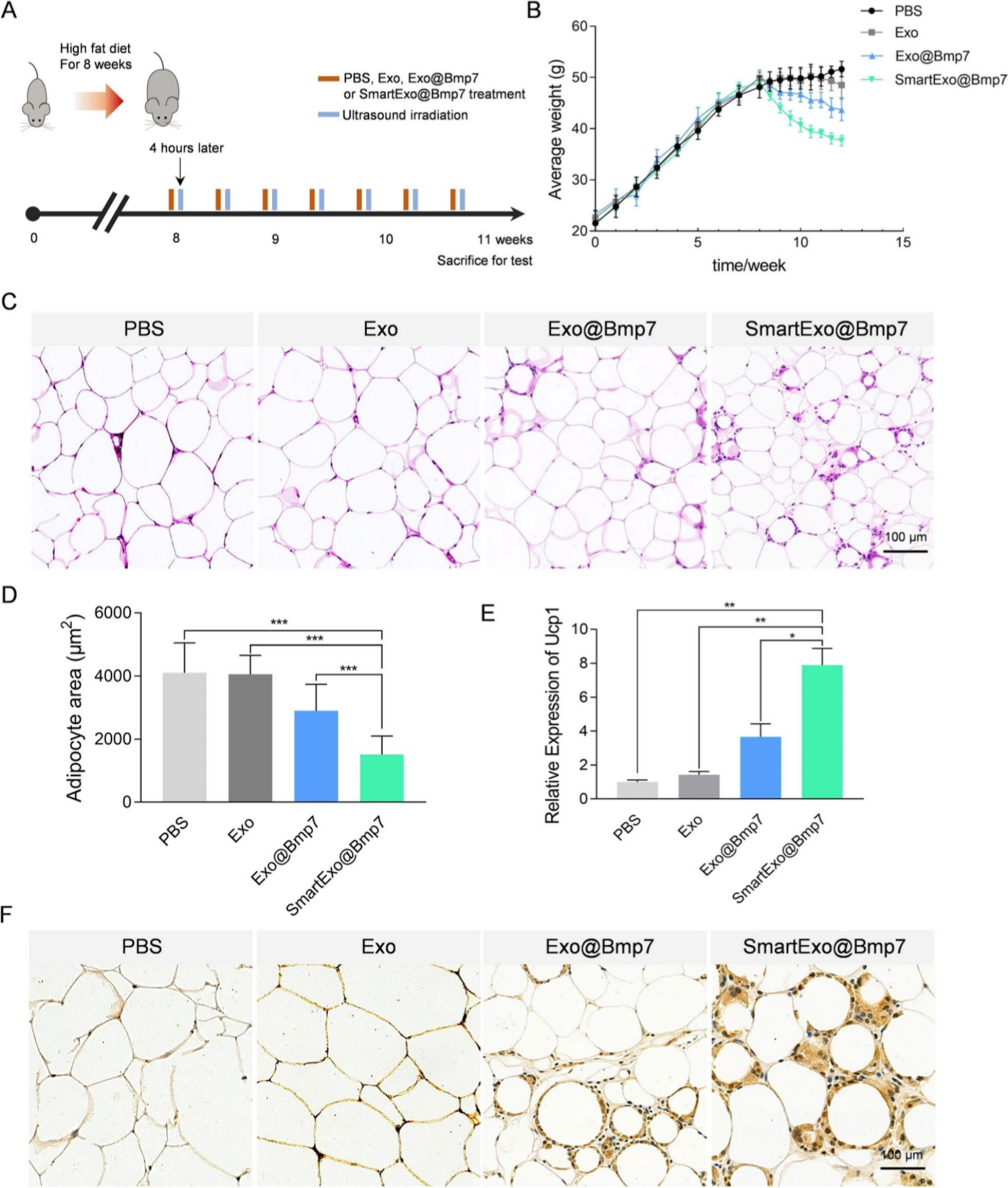
The efficiency of the US triggered a smart exosome-based system for *Bmp7 delivery* for OAT browning. **A** Schematic representation of the experimental design. **B** Each group's average weight was observed from the high-fat diet till the end of the treatment period. **C** HE staining images of OAT from mice after *i.v* treatment. **D** Adipocyte area for HE staining images. **E** *Ucp1 expression level* in OAT from mice after *i.v* treatment. **F** Images of *Ucp1* staining of a section of OAT depots from mice after *i.v* treatments. Reprinted with permission from Ref. [121]. Copyright © 2023 BioMed Central Ltd

CP05-TK-mPEG switchable stealth core-decorating exosomes were developed and loaded with Chlorin e6 (Ce6) and bone Bmp7 mRNA. This smart system can locally degrade by ultrasound (US), resulting in precise delivery of Bmp7 mRNA to the omental adipose tissue (OAT) of obese mice (Fig. 4) [97]. While this innovative strategy has shown promise in tumor models, further studies are needed to confirm its applicability for cancer treatment. By utilizing additional physical influences such as ultrasonic irradiation and lasers, the timing of the release of genetically engineered exosomes can be controlled, thus providing temporal control over the delivery of therapeutic payloads.

### Genetic modification of exosomes

mRNA vaccine therapy is a powerful and attractive immunotherapeutic platform against tumors because of its versatility, safety, specificity, high potency, large-scale and rapid development capability and low manufacturing cost. To deliver mRNA-based personalized cancer vaccines, OMV were genetically modified with RNA-binding protein L7Ae and lysosomal-escape protein Listeria monocytogenes hemolysin O (OMV-LL). The developed nanoparticle system presented “plug-and-play” functionality, allowing the development of personalized tumor vaccines based on specific tumor antigens. When exosome-carrying ovalbumin or ADPGK mRNA was injected subcutaneously into mice, it significantly inhibited melanoma progression.

To specifically target exosomes to tumor cells, HEK293 cells were transfected with a plasmid containing the coding sequence for the EVHB chimeric protein. This protein consists of a high-affinity anti-HER2 scFv antibody that can be displayed on the surface of exosomes. In addition, these cells were transfected with a plasmid carrying a genetic code for a modified E. coli enzyme called HChrR6, which can convert the prodrug 6-chloro-9-nitro-5-oxo-5H-benzo-(a)-phenoxazine (CNOB) to the cytotoxic drug MCHB. In vivo, administration of these engineered exosomes resulted in the efficient expression of HChrR6 in tumor cells. As a result, the growth of BT474 xenografts was almost completely arrested after the prodrug treatment [123].

The EXOtic (exosome transfer to cell) device was developed for loading mRNA into parental cells. As the Fig. 2A have shown exosomes were engineered by combining catalase mRNA to a C/D box for efficient exosomal targeting. The developed system carrying RVG-Lamp2b successfully crossed the BBB and delivered catalase mRNA. This reduced neuroinflammation induced by 6-OHDA and neurotoxicity in a Parkinson's mouse model [101].

A suitable transfection reagent is used to transfect different cells with the mRNA of interest. The mRNA-transfection reagent complex is then added to the cells and incubated for an appropriate duration. Supernatant containing the exosomes is collected, and after centrifugation, an exosomes pellet containing mRNA is obtained (Fig. 3).

**Fig. 5:**
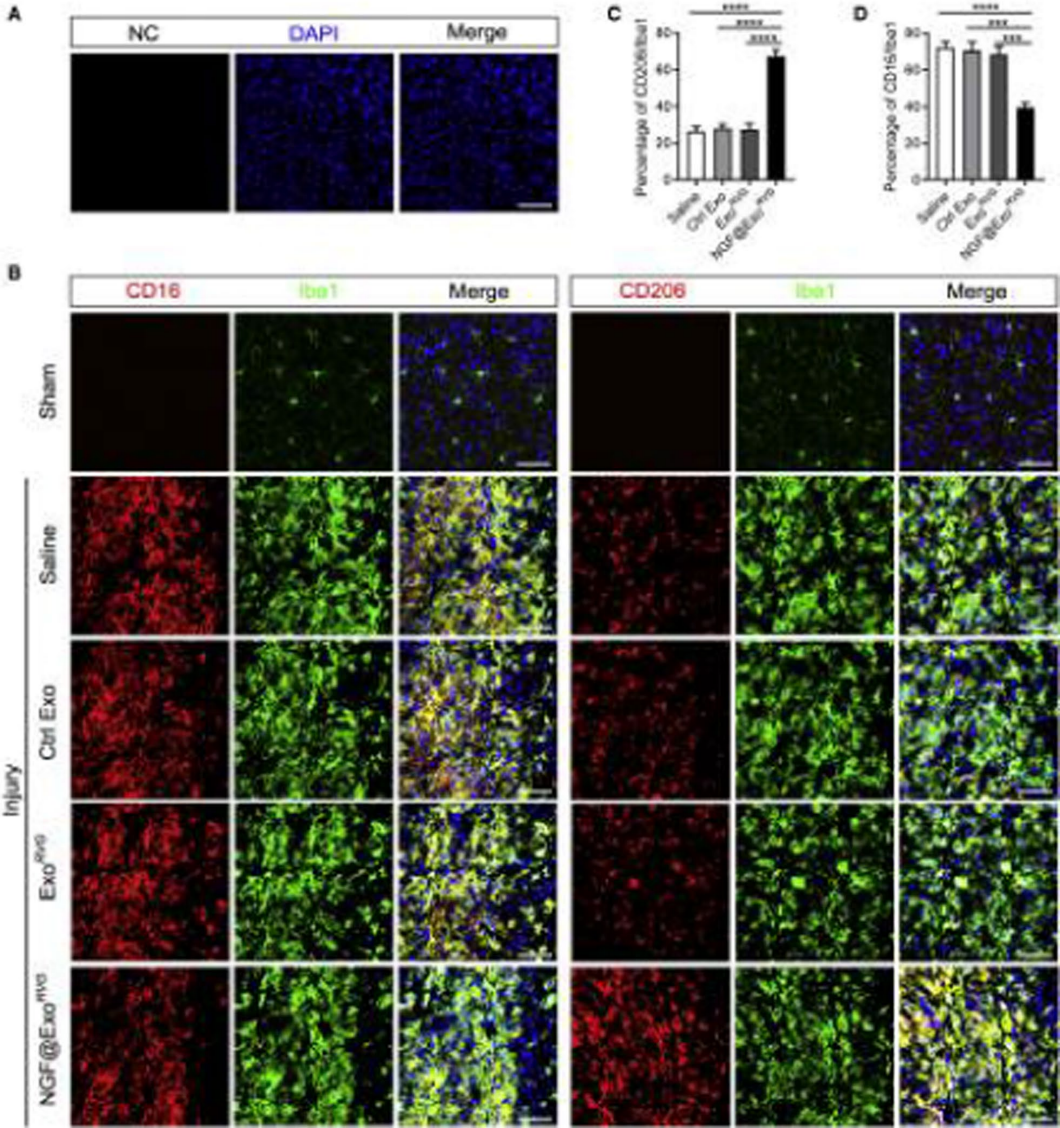
Role of NGF@ExoRVG on Microglia Polarization in brain injury. **A** Immunofluorescence images confirming the specificity of immunostaining via secondary antibody negative control (NC) **B** Representative immunofluorescence images of different antibodies staining in the ischemic cortex of different treatments. NGF@ExoRVG showed a significant decrease in CD16 expression and a remarkable increase in the CD206 expression compared with other groups **C**, **D** Cell quantification of the % age of CD206 and CD16 in the ischemic region. Reprinted with permission from Ref. [124], Copyright © 2023 Elsevier B.V

Exosomes were modified with RVG peptides to specifically target neurons and loaded with NGF to produce the system called NGF@ExoRVG. When administered systemically, the NGF@ExoRVG exhibited effective delivery into the ischemic cortex. Bursts release of NGF protein was observed, and the system exhibited a high degree of stability on storage, allowing it to function effectively in vivo for extended periods (Fig. 5) [124]. The findings suggest that inflammation can be reduced by modulating microglia polarization, highlighting this system's potential for treating stroke.

Phage MS2 system and LAMP-2B were used to modify exosomes. This system demonstrated the ability to specifically load and deliver anti-CD3/CD28 scFv and CAR mRNA. It can be used for the ex vivo production of CAR T cells with the ability to kill tumor cells. The results suggested that this engineered exosome delivery system has great potential for converting primary T cells into CAR T cells and provides a new approach for the in vivo induction of CAR T cells [106].

Cytosine deaminase (CD)-uracil phosphoribosyltransferase (UPRT) was transfected into donor cells to modify them genetically. Microvesicles (MV) containing CD-UPRT mRNA/protein were isolated three days later. The injection of this system directly into schwannomas resulted in significantly high expression of functional proteins and inhibition of tumor growth following systemic treatment with the prodrug 5-FC. This study demonstrates the effectiveness of MV in delivering therapeutic proteins and mRNAs to treat various diseases [125].

Application of KK-(EK)4-lipids to exosomes and lipid nanoparticles encapsulating mRNA increased cellular association and enhanced protein expression in vitro for modified exosomes and mRNA-LNPs, respectively, compared to the unmodified nanocarrier. These findings demonstrate the potential of KK-(EK)4-lipid for liposomes, exosomes and mRNA-LNP intracellular delivery, proving its versatility and promise in this regard [126].

**Fig. 6:**
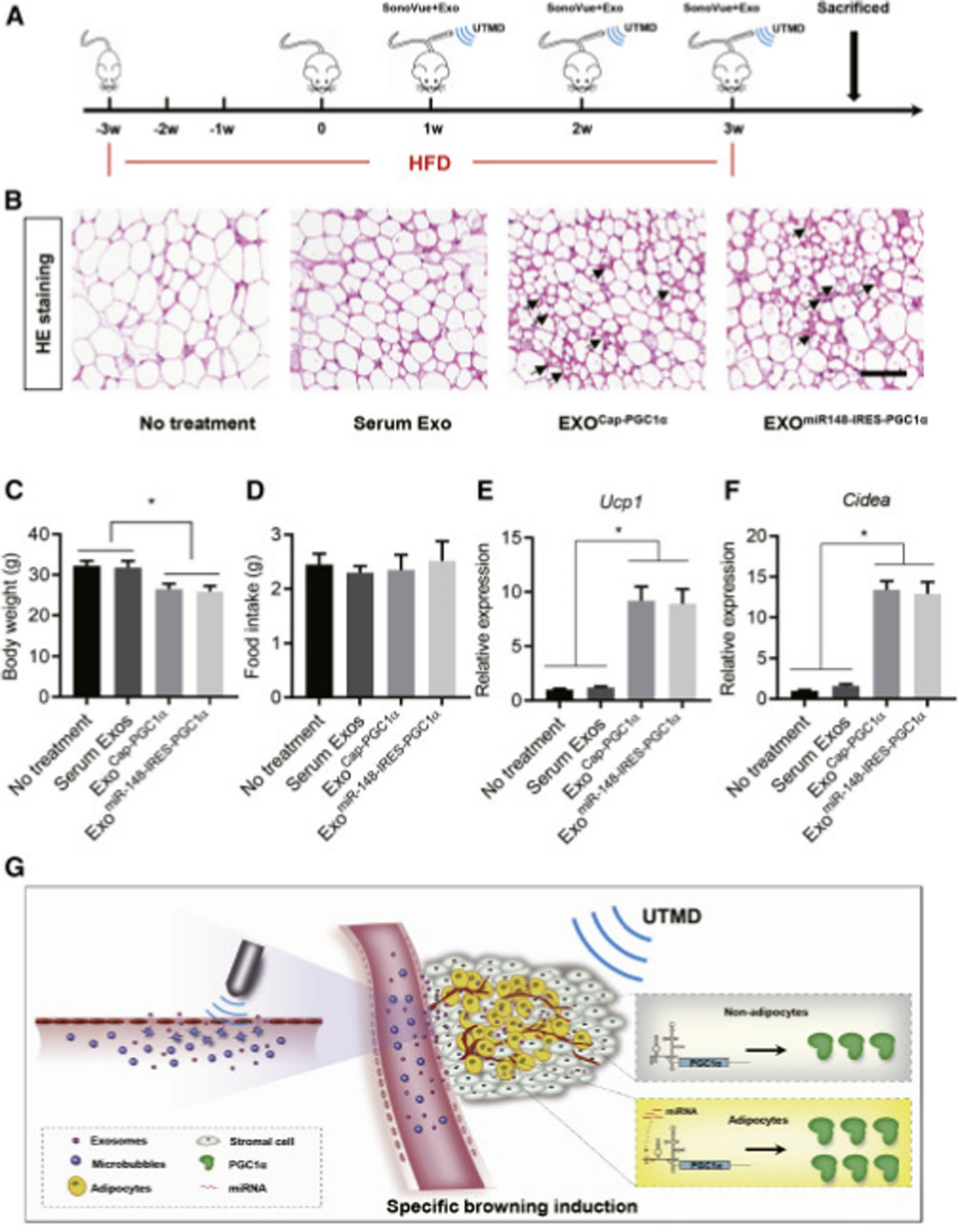
Efficient delivery of mIRES-PGC1α mRNA promotes browning in mouse adipose tissues. **A** Schematic illustration of the experimental procedure. Mice were fed with a high-fat diet for 3 weeks, followed by exosome delivery with the aid of UTMD. The exosome delivery was performed once a week for 3 continuous weeks. Mice were sacrificed at the end of experiments for histology and gene expression. **B** H&E staining of the adipose tissue in mice treated as indicated. Smaller browning adipocytes are indicated by arrows. Scale bar, 50 μm. Data shown are representative of five to seven mice in each group. **C** Body weight in mice treated as indicated. Data are expressed as mean ± SEM of seven mice in each group. ∗ p < 0.05 by one-way ANOVA. **D** Food intake in mice treated as indicated. Data are expressed as mean ± SEM of seven mice in each group. ∗ p < 0.05 by one-way ANOVA. **E** and **F** qPCR analysis of Ucp1 (**E**) and Cidea (**F**) expression in adipose tissue. Gapdh served as internal control. Data are expressed as mean ± SEM of three mice in each group. ∗ p < 0.05 by one-way ANOVA. **G** Schematic illustration of the study. Reprinted with permission from Ref. [97]. Copyright © 2023 BioMed Central Ltd

Exosomes were utilized to transport PGC1α mRNA, which is responsive to miR-148a and specific to adipose tissue. Through microvesicle disruption guided by ultrasound, the system achieved successful delivery to the targeted adipose tissue. This method effectively enhanced the expression of PGC1α protein, particularly in mouse adipose tissue, while reducing expression in other tissues such as the lung. As a result, the overall therapeutic effect was improved while minimizing unintended effects on non-target tissues (Fig. 6) [121].

A specialized EV system known as EVTx was created to transport GSDMD-N mRNA for the purpose of enhancing cancer immunotherapy. The EVs underwent modifications with Ce6 and HER2 antibodies to improve targeting capabilities. Upon delivery to HER2-positive breast tumor cells, this engineered system successfully reversed translational repression. Furthermore, it exerted an additional therapeutic mechanism by triggering apoptosis in cancer cells through the application of sonodynamic therapy [127].

## Applications of exosomes in the delivery of mRNA

Therapeutic use of mRNA inspired a substantial hope against striving various incurable diseases. mRNA as a therapeutic agent, recent breakthroughs in nanotechnology and molecular sciences have made it possible to produce practically any functional protein or peptide in the human body. Recent research has highlighted the potential of exosomes as a viable carrier for mRNA-based drug delivery owing to their biocompatibility, minimal immunogenicity, toxicity, and inherent ability to cross the BBB, thus making them promising nanocarriers [87, 128]. Exosomes loaded with mRNA drugs may be passively transported throughout the body or targeted through surface engineering [87]. Internalization of exosomes at target cells may occur predominantly by endocytosis, membrane fusion, or receptor-mediated uptake [129].

Furthermore, exosomes derived from specific cell lines, such as dendritic cells, natural killer cells, or stem cells, can encapsulate mRNA to produce proteins that modulate the expression of disease-related proteins in conditions like glioblastoma, breast cancer, COVID, Parkinson's disease, leukemia, kidney disease, and infectious diseases. In recent years, intradermal delivery of mRNA-loaded exosomes using microneedles has shown promise in efficiently secreting specific proteins [130–132].

### CNS diseases

To treat Parkinson's disease, exosomes were produced from the HEK-293 cell line and used as potential carriers for delivering RNA drugs. These catalase mRNA-loaded exosomes were specifically targeted to the brain. Selective delivery of catalase to the brain hampered oxidative damage to neurons, the main contributor to neuronal cell death. The study results revealed a reduction of 6-OHDA-induced neuroinflammation in Parkinson’s disease [133].

### Cancer

Researchers developed exosomes derived from dendritic cells and HEK-293 cells, which were loaded with mRNA encoding the HChrR6 enzyme. When the designed exosomes were delivered to HER2 + human breast cancer cells, they exhibited selective killing and almost complete suppression of breast cancer in a mouse model. The target killing was achieved by HchrR6-induced conversion of prodrug CNOB into active drug 9-p amino-6-chloro-5H-benzo[a]phenoxazine-5-one (MCHB) in tumors [122].

Forterre et al*.* developed in vitro transcribed (IVT) HChrR6 mRNA-exosomes and revealed selective uptake by HER2 cancer cells without toxicity to other cells and very insignificant compromise to bone marrow. CB1954, as a prodrug, was employed, which showed better suppression of HER2 breast cancer cells after conversion into an active drug [134]. Exosome-based mRNA delivery has also demonstrated therapeutic value in leukemia in addition to breast cancer. RBC-derived exosomes loading Cas9 mRNA system was developed for treating acute myeloid leukemia (ALL). The targeted delivery of Cas9 RNA showed a robust reduction in miR-125a and miR-125b expressions, well-known oncogenic miRNAs in leukemia [92].

Yang et al*.* modified CD47-derived exosomes by incorporating a glioma targeting peptide at the N terminus. This modification allowed for selective uptake of tumor suppression gene, phosphatase and tensin homolog (PTEN) cargo by glioma cells. As a result, the proliferation of tumor cells was effectively inhibited [110].

Mizrak et al*.* engineered genetically modified exosomes derived from HEK-293 cells, which carried mRNA/protein encoding suicide cytosine deaminase (CD) and uridine phosphoribosyl transferase (UPRT) to treat nerve sheath tumors (schwannomas). The study revealed that CD converted the prodrug (5-FC) into its active form (5-FU), and consequentially, the DNA synthesis was inhibited, leading to the apoptosis of cancer cells [122].

### Infectious diseases

Popowski et al. developed an inhalable vaccine utilizing lung-derived exosomes for mRNA and protein cargos. They indicated that Lung-Exo exhibited pronounced mRNA translation, protein expression, and improved cargo retention in bronchioles and parenchyma, suggesting better therapeutic efficacy against severe acute respiratory syndrome coronavirus -2 (SARS-COV-2) [135].

Another study indicated the development of an exosomes-derived vaccine loaded with mRNA encoding N and S spike proteins of the immunogenic SARS-CoV-2 virus. The designed vaccine manifested improved and prolonged humoral and cellular immunity against the viral N and S proteins with minimal untoward effects compared to the available SARS-CoV-2 vaccine [117].

### Metabolic disorders

Localized delivery of smart Bmp7 mRNA was achieved using decorated exosomes for the treatment of obesity. The study results revealed that localized US-assisted delivery of Bmp7 elicited efficient browning of OAT and proved as a promising anti-obesity therapy [97]. Zhao et al*.* engineered exosomes using adipose-derived stem cells (ADSC) to treat obesity. The smart exosomes reduced WAT inflammation, improved energy expenditure and resilience to obesity progression [136].

### Cardiovascular diseases

Exosomes functionalized with RVG and loaded with NGF-mRNA were developed. On systemic administration, the system reduced inflammation and promoted cell survival, thus exhibiting the promising therapeutic potential for stroke. This system can also be proved effective for CNS diseases [124]. Potential applications of exosomes mediated mRNA delivery are shown in Table 2.

**Table 2:** Potential applications of exosomes in the mRNA delivery

Disease	Source cell line	Targeted moiety	Outcome	References
Cancer	Breast cell	HchrR6 mRNA	Growth inhibition of HER2-positive breast cancer in human	[123]
	SKOV3 human ovarian cancer cells	CRISPR/Cas9	Targeted accumulation in ovarian cancer and induction of apoptosis of cancer cells	[137]
	Mesenchymal stromal cells (MSCs) from adipose tissue	yCD::UPRT mRNA	Drastic reduction in tumor burden	[138]
	Dental pulp mesenchymal stem cells	yCD::UPRT mRNA tagged with iron	Caused tumor cell death	[139]
Infectious diseases	Lung cells	mRNA	Deposition of mRNA in bronchioles and parenchyma to treat lung issues associated with COVID	[135]
Metabolic disorders	Adipose cells	Bmp7	Improved anti-obesity potential	[97]
	HEK293T cells	miR-148a (an adipose relatively specific miRNA)-responsive PGC1α mRNA	Pronounced fat browning in the adipose tissue and less off-target effects	[121]
Parkinson’s disease	HEK-293 T cells	Catalase	Potent neuroprotective effect	[101]

## Conclusion and prospective

The potential of exosomes as a carrier has sparked immense interest over several years. Exosomes possess several characteristics that make them ideal carriers for drug delivery, *e.g.*, biocompatibility, low immunogenicity, ability to cross the BBB and targeted delivery capabilities. They are promising in delivering a variety of payloads such as siRNA, miRNA and mRNA. mRNA therapies have an edge over other therapies owing to their versatility, as they can be modified to produce a variety of therapeutic proteins, making them a flexible system for treating different diseases. Moreover, they have the potential for rapid development, thus permitting a prompt response to emerging diseases or new targets. From cancer to COVID-19, exosomes present a great possibility to improve the efficacy of mRNA in treating a wide range of disorders.

However, several challenges remain that need to be addressed in order to advance the clinical application of exosome-mediated drug delivery. The methods for isolating, purifying, and loading exosomes need to be optimized. Advanced and well-defined techniques are required to characterize them more effectively. The optimal dosage of mRNA delivered via exosomes should be determined. Furthermore, efforts should be made to enhance the targeting and tissue specificity of exosomes to ensure successful delivery to the desired cells or tissues. Currently, the use of nanoparticles for mRNA therapy are still limited to the laboratory scale, and further research is needed to advance the use of these techniques at the patient bedside. Finally, the potential immunogenicity of exosome-mediated mRNA delivery needs to be thoroughly investigated to ensure their clinical viability.

Overcoming these challenges will be pivotal for successfully translating exosome-mediated mRNA delivery into clinical applications, revolutionizing the mRNA drug field. A series of preclinical studies, including pharmacodynamic and toxicity evaluations, are necessary to address potential safety concerns and minimize potential side effects. To corroborate the successful commercialization of exosome-based therapeutics, scientists and commercial manufacturers must invest efforts in resolving technical and logistical issues that will pave the way for the widespread adoption of exosome-based therapies. In the near future, remarkable advances in exosome engineering and cargo-loading approaches are expected, enabling precise and targeted delivery of therapeutic mRNAs for the treatment of various diseases. Using autologous exosomes for therapeutic drug delivery is expected to mitigate uncertain immunogenicity issues in the clinical setting. Continued research and development in exosome-mediated mRNA delivery will intensely impact healthcare and biomedical research, opening up new possibilities for effective therapies.

## Data Availability

No datasets were generated or analysed during the current study.
